# Contribution of the Collagen-Binding Proteins of *Streptococcus mutans* to Bacterial Colonization of Inflamed Dental Pulp

**DOI:** 10.1371/journal.pone.0159613

**Published:** 2016-07-21

**Authors:** Ryota Nomura, Yuko Ogaya, Kazuhiko Nakano

**Affiliations:** Department of Pediatric Dentistry, Division of Oral Infections and Disease Control, Osaka University Graduate School of Dentistry, Suita, Osaka 565–0871, Japan; LSU Health Sciences Center School of Dentistry, UNITED STATES

## Abstract

*Streptococcus mutans* is a major pathogen of dental caries. Collagen-binding proteins (CBPs) (approximately 120 kDa), termed Cnm and Cbm, are regarded as important cell surface antigens related to the adherence of *S*. *mutans* to collagenous tissue. Furthermore, CBP-positive *S*. *mutans* strains are associated with various systemic diseases involving bacteremia, such as infective endocarditis. Endodontic infection is considered to be an important cause of bacteremia, but little is known regarding the presence of *S*. *mutans* in dental pulp tissue. In the present study, the distribution and virulence of *S*. *mutans* in dental pulp tissues were investigated by focusing on CBPs. Adhesion and invasion properties of various *S*. *mutans* strains were analyzed using human dental pulp fibroblasts (HDPFs). CBP-positive strains had a significantly higher rate of adhesion to HDPFs compared with CBP-defective isogenic mutant strains (*P*<0.001). In addition, CBP-positive strains induced HDPF proliferation, which is a possible mechanism related to development of hyperplastic pulpitis. The distribution of *S*. *mutans* strains isolated from infected root canal specimens was then analyzed by PCR. We found that approximately 50% of the root canal specimens were positive for *S*. *mutans*. Approximately 20% of these strains were Cnm-positive, while no Cbm-positive strains were isolated. The Cnm-positive strains isolated from the specimens showed adhesion to HDPFs. Our results suggest that CBP-positive *S*. *mutans* strains exhibit high colonization in dental pulp. This could be a possible virulence factor for various systemic diseases.

## Introduction

*Streptococcus mutans* is a major pathogen of dental caries and is also regarded as a causative agent of infective endocarditis [1]. Collagen-binding proteins (CBPs) of approximately 120 kDa, termed Cnm and Cbm, in *S*. *mutans* have been characterized as LPXTG-anchored cell surface proteins [2, 3]. Cnm and Cbm have a frequency of distribution in *S*. *mutans* oral strains of approximately 10–20% and 2%, respectively [3, 4]. In our previous study, bacterial DNA of CBP-positive *S*. *mutans* strains was detected in cardiovascular specimens at a high frequency [5]. Additionally, *in vitro* and *in vivo* findings have demonstrated that these strains are associated with the pathogenesis of infective endocarditis [5]. More recently, we reported that these CBP-positive strains are possible virulence factors associated with cerebral hemorrhage, ulcerative colitis, non-alcoholic steatohepatitis, and IgA nephropathy [6–9].

With progression of dental caries, enamel demineralization occurs, exposing dentin tissue [10]. CBP-positive *S*. *mutans* strains have an advantage for attachment to dentin because type I collagen is the main organic component, comprising 85–91% of the organic matrix [2, 11]. In a recent study that used an *ex vivo* tooth binding model, a Cnm-positive strain showed enhanced binding to collagenous dentin [12]. Bacteria harbored deep in dentin caries are considered to be the primary source of endodontic infection [13]. This is because most species that are detected in carious dentin are the same as those found in infected root canals [14]. These endodontic bacteria are thought to invade the bloodstream, resulting in bacteremia, which is the first step in the pathogenesis of systemic diseases induced by oral bacteria [15]. Dental procedures, and even daily tooth brushing, may cause translocation of microorganisms from the oral cavity into the bloodstream [16]. Additionally, treatment of an infected root canal, which may be a reservoir for oral bacteria, increases the risk of bacteremia [17]. However, few reports have described the pathogenicity of *S*. *mutans* in inflamed pulp.

In the present study, we focused on the pathogenicity of CBP-positive *S*. *mutans* strains in relation to human dental pulp fibroblast cells (HDPFs). The distribution of CBP-positive *S*. *mutans* strains in inflamed pulp specimens, and adhesion and invasion properties of isolated strains were investigated with a primary focus on CBPs.

## Materials and Methods

### *S*. *mutans* strains and growth conditions

This study was conducted in full adherence to the Declaration of Helsinki. The study protocol was approved by the Ethics Committee of the Osaka University Graduate School of Dentistry (approval number: H23-E1). Prior to the collection of specimens, the subjects or their guardian (when the subjects were children) were informed of the study contents by use of written forms, as well as verbal explanations. Written informed consent for participation was obtained. The Cnm-positive *S*. *mutans* strain TW295 (serotype *k*) and its Cnm-defective mutant TW295CND [18], as well as the Cbm-positive strain NN2193-1 (*k*) and its Cbm-defective mutant NN2193-1CBD [3], were used. In addition, the We also used CBP-negative strains MT8148 (*c*) and NN2004 (*c*). Additionally, the Cnm-positive strain TW871 (CBP+), its Cnm-defective mutant TW871CBD [19], and TW871PD, an isogenic mutant of TW871 without protein antigen (PA) expression [18], were used. *S*. *mutans* clinical strains isolated from inflamed dental pulp were used (S1 and S2 Tables). All strains were confirmed to be *S*. *mutans* based on their biochemical properties and observation of rough colony morphology on Mitis-salivarius agar (Difco Laboratories, Detroit, MI, USA) plates containing bacitracin (0.2 U/mL; Sigma-Aldrich Co., St. Louis, MO, USA) and 15% (wt/vol) sucrose (MSB agar). For growth conditions, all strains were cultured overnight in brain heart infusion broth (Difco Laboratories) from our laboratory stock. Bacterial cells were collected by centrifugation and washed by phosphate-buffered saline (PBS). If necessary, bacterial numbers were adjusted for each assay, as described in the subsections of “Adhesion to and invasion of HDPFs”, “Cell proliferation assay” and “Collagen-binding properties of clinical strains”. Isogenic mutant strains were cultured in brain heart infusion broth with erythromycin added to a final concentration of 10 μg/mL.

### Cell cultures

Prior to collection of specimens, the subjects or their guardians were informed of the study contents, and written informed consent was obtained from all of the participants. Normal human dental pulp tissue was obtained from a non-carious primary canine of an 8-year-old boy at the time of an expedient pulpectomy for orthodontic treatment. In addition, a non-carious third molar of a 50-year-old man that was extracted because of pericoronitis was also obtained and HDPFs were established from explant cultures of the pulp tissue, as previously described [20]. The explants were cultured in EBM-2 (Lonza, Walkersville, MD, USA) at 37°C in a water-saturated atmosphere of 95% air and 5% CO_2_. HDPFs obtained in this manner were maintained and used within five to nine passages.

### Adhesion to and invasion of HDPFs

The adhesion and invasion properties of *S*. *mutans* strains with HDPFs were evaluated using a previously described method [21, 22], with some modifications. Approximately 1×10^5^ HDPFs were seeded in parallel wells of 24-well tissue culture plates (Costar^®^, Corning Inc., Corning, NY, USA). Prior to infection, the wells were washed three times with PBS and antibiotic-free medium was added. HDPFs were infected by addition of 1×10^7^ colony-forming units (CFU) of *S*. *mutans* strains in antibiotic-free medium to the wells. For adhesion assays, the medium was removed after 1.5 h of aerobic incubation and infected cells were washed three times with PBS, followed by addition of sterile distilled water to disrupt the cells. For invasion assays, the medium was removed after 2 h of aerobic incubation and infected cells were washed three times with PBS. Medium containing penicillin (50 μg/mL) and gentamycin (300 μg/mL) was then added and incubation was performed for 3 h, after which sterile distilled water was added to disrupt the cells. For the adhesion and invasion assays, dilutions of cell lysates infected with *S*. *mutans* were plated onto MSB plates and cultured at 37°C for 2 days. The adherence and invasion rates were determined by the ratio of resuspended to infected cells. Data are expressed as the mean ± standard deviation of triplicate experiments.

### Fluorescence microscopy

To confirm adhesion and invasion of bacteria visually, a double-fluorescence technique was used for observation with confocal scanning laser microscopy, as previously described [5]. The infected cells were washed three times with PBS, fixed with 3% paraformaldehyde (Wako Pure Chemical Industries, Osaka, Japan) for 10 min, and washed with PBS. They were then incubated with rabbit anti-Cnm serum [3] diluted to 1:500 with PBS–0.5% bovine serum albumin for 60 min at room temperature. Following incubation, culture dishes were washed three times with PBS and incubated with Alexa Fluor 555-conjugated goat anti-rabbit immunoglobulin G (Molecular Probes^®^, Life Technologies Co., Eugene, OR, USA), which was diluted to 1:500 with PBS–0.5% bovine serum albumin for 30 min at room temperature to visualize attached bacteria. The HDPFs were then permeabilized by adding 0.4% Triton X-100 solution for 5 min and actin filaments were stained with Alexa Fluor 488 conjugated to phalloidin (Molecular Probes^®^) for 30 min at room temperature to visualize the cellular cytoskeleton. Nuclei were stained with 4′, 6-diamidino-2-phenylindole, dihydrochloride solution (Wako Pure Chemical Industries). Culture dishes were examined by confocal scanning laser microscopy using a TCS-SP5 microscope (Leica Microsystems GmbH, Wetzlar, Germany), as well as a DMI6000 B fluorescence microscope (Leica) and a 63× oil immersion objective.

### Cell proliferation assay

Methyl tetrazolium (MTT) assays were performed using a previously described method [23], with some modifications. Briefly, 1×10^5^ HDPF cells were cultured in 24-well culture plates and infected by addition of 1×10^7^ CFU of each tested *S*. *mutans* strain in antibiotic-free medium to the wells. The medium was removed after 6 h of aerobic incubation. Infected cells were washed three times with PBS, and then 100 μL of an MTT (Sigma-Aldrich Co.) solution (5 mg/mL) was added to each well with 1 μL of medium, and cells were incubated at 37°C for 4 h. Next, 1 μL of 0.04 N HCl in isopropanol was added and mixed thoroughly to dissolve the dark blue crystals. The plates were allowed to stand at room temperature overnight. Readings were performed using a Benchmark Plus microplate spectrophotometer (Bio-Rad Laboratories, Hercules, CA, USA) at a wavelength of 595 nm. Data are shown as the mean of four determinations.

### Isolation of *S*. *mutans* in inflamed dental pulp specimens

Prior to collection of the specimens, the subjects or their guardians were informed of the study contents and written informed consent was obtained from all of the participants. The subjects were 64 children and adolescents (age range: 1–20 years) who visited Osaka University Dental Hospital from February 2013 to September 2014 to receive root canal treatment under local anesthesia because of severe childhood caries (n = 49), trauma (n = 12), or fracture of the central cusp (n = 3). Root canal specimens were collected using sterile root canal instruments and stored in sterile saline in sterile plastic tubes. Specimens were streaked onto Mitis-salivarius agar (Difco Laboratories) plates containing bacitracin (0.2 U mL-1; Sigma-Aldrich Co.), as well as 15% sucrose (MSB agar). Five colonies from the plates of each subject were selected and grown in brain heart infusion broth (Difco Laboratories).

### Molecular methods for determination of the serotype and presence of genes encoding CBPs of *S*. *mutans* strains

Bacterial DNA was extracted using a previously described method [24]. Briefly, bacterial cells were collected and incubated with 62.5 μL of lysozyme chloride from chicken egg white (2.0 mg/mL; Sigma-Aldrich Co.) and 0.25 μL of lysozyme hydrochloride from chicken egg white (10 mg/mL; Wako Pure Chemical Industries) for 90 min at 37°C. Genomic DNA was then extracted using 600 μL of Cell Lysis solution (Qiagen, Düsseldorf, Germany) and incubated at 80°C for 5 min, followed by addition of 3 μL of RNase A (10 mg/mL; Qiagen) to the mixture and incubation at 37°C for 30 min. In addition, 200 μL of Protein Precipitation solution (Qiagen) was added and vortexed vigorously for 20 min, and then centrifuged at 10,000 × g for 3 min. The supernatant was combined with 600 μL of isopropanol (Wako Pure Chemical Industries) and centrifuged. The precipitate was then resuspended with 70% ethanol (Wako Pure Chemical Industries), centrifuged, combined with 100 μL of DNA hydration solution (Qiagen), and stored as a DNA extract. Confirmation of *S*. *mutans*, serotype determination, and collagen-binding gene detection were carried out by PCR using TaKaRa Ex Taq polymerase (TAKARA BIO, Shiga, Tokyo, Japan) with *S*. *mutans*-, serotype-, *cnm*-, and *cbm*-specific sets of primers [3, 4, 24–26] (Table 1), template DNA, and 1.5 mM of MgCl_2_, according to the supplier’s protocols. Amplification was performed using the following parameters. To detect *S*. *mutans*, 30 cycles of a denaturing step at 98°C for 10 s, and primer annealing and extension steps at 70°C for 1 min were performed. To detect the *cnm* and *cbm* genes, we performed initial denaturation at 95°C for 4 min, and then 30 cycles consisting of 95°C for 30 s, 60°C for 30 s, and 72°C for 2 min, with a final extension at 72°C for 7 min. The PCR products were subjected to electrophoresis in 1.5% or 0.7% agarose gel-Tris-acetate-EDTA buffer. The gel was stained with 0.5 μg of ethidium bromide per mL and photographed under ultraviolet illumination.

**Table 1:** PCR primers used in the present study.

Primer set	Purpose	Sequence (5′–3′)	Amplified size	Reference
MKD-F	*S*. *mutans* detection	GGC ACC ACA ACA TTG GGA AGC TCA GTT	433	25
MKD-R		GGA ATG CCG ATC AGT CAA CAG GAT		
SC-F	Serotype *c* determination	CGG AGT GCT TTT TAC AAG TGC TGG	727	26
SC-R		AAC CAC GGC CAG CAA ACC CTT TAT		
SE-F	Serotype *e* determination	CCT GCT TTT CAA GTA CCT TTC GCC	517	26
SE-R		CTG CTT GCC AAG CCC TAC TAG AAA		
SE-F	Serotype *f* determination	CCC ACA ATT GGC TTC AAG AGG AGA	316	26
SE-R		TGC GAA ACC ATA AGC ATA GCG AGG		
CEFK-F	Serotype *k* determination	ATT CCC GCC GTT GGA CCA TTC C	294	27
K-R		CCA ATG TGA TTC ATC CCA TAC C		
Cnm-1F	*cnm* amplification	GAC AAA GAA ATG AAA GAT GT	1728	4
Cnm-1R		GCA AAG ACT CTT GTC CCT GC		
Cbm-1F	*cbm* amplification	GAC AAA CTA ATG AAA TCT AA	1814	3
Cbm-1R		GCA AAA ACT GTT GTC CCT GC		

### Collagen-binding properties of clinical strains

Collagen-binding properties were evaluated according to a previously described method [28], with some modifications. Type I collagen (collagen [type I] in 0.1 N acetic acid; Sigma-Aldrich Co.) was coated onto 96-well plates (Tissue Culture Plate; Beckton Dickinson Ltd., Franklin Lakes, NJ, USA) overnight at 4°C. The plates were then washed three times with PBS and blocked for 1.5 h with 5% bovine serum albumin in PBS. The wells were washed again with PBS containing 0.01% Tween 20. Cells from overnight cultures of *S*. *mutans* grown in brain heart infusion broth were collected by centrifugation. Bacterial numbers were adjusted with PBS, and then added to the coated wells (2×10^9^ CFU per well). After a 3-h incubation at 37°C, adherent cells were washed three times with PBS, and then fixed with 200 μL of 25% formaldehyde at room temperature for 30 min. After an additional three washes with PBS, adherent cells were stained with 200 μL of 0.05% crystal violet (Wako Pure Chemical Industries) in water for 1 min. These cells were washed three times with PBS and the dye was dissolved by adding 7% acetic acid (200 μL), after which density measurements were performed at a wavelength of 595 nm. Results for each strain are expressed as the values when the properties of TW871 were defined as 100%. Data are expressed as the mean ± standard deviation of three independent experiments using three wells for each sample.

### Statistical analyses

Statistical analyses were performed using the computational software package StatView 5.0 (SAS Institute Inc., Cary, NC, USA). Intergroup differences for adhesion, invasion, and proliferation rates were analyzed using the Student’s t-test. A *P* value of less than 0.05 was considered to be statistically significant.

## Results

### *S*. *mutans* adhesion to and invasion of human pulp cells

**Fig 1:**
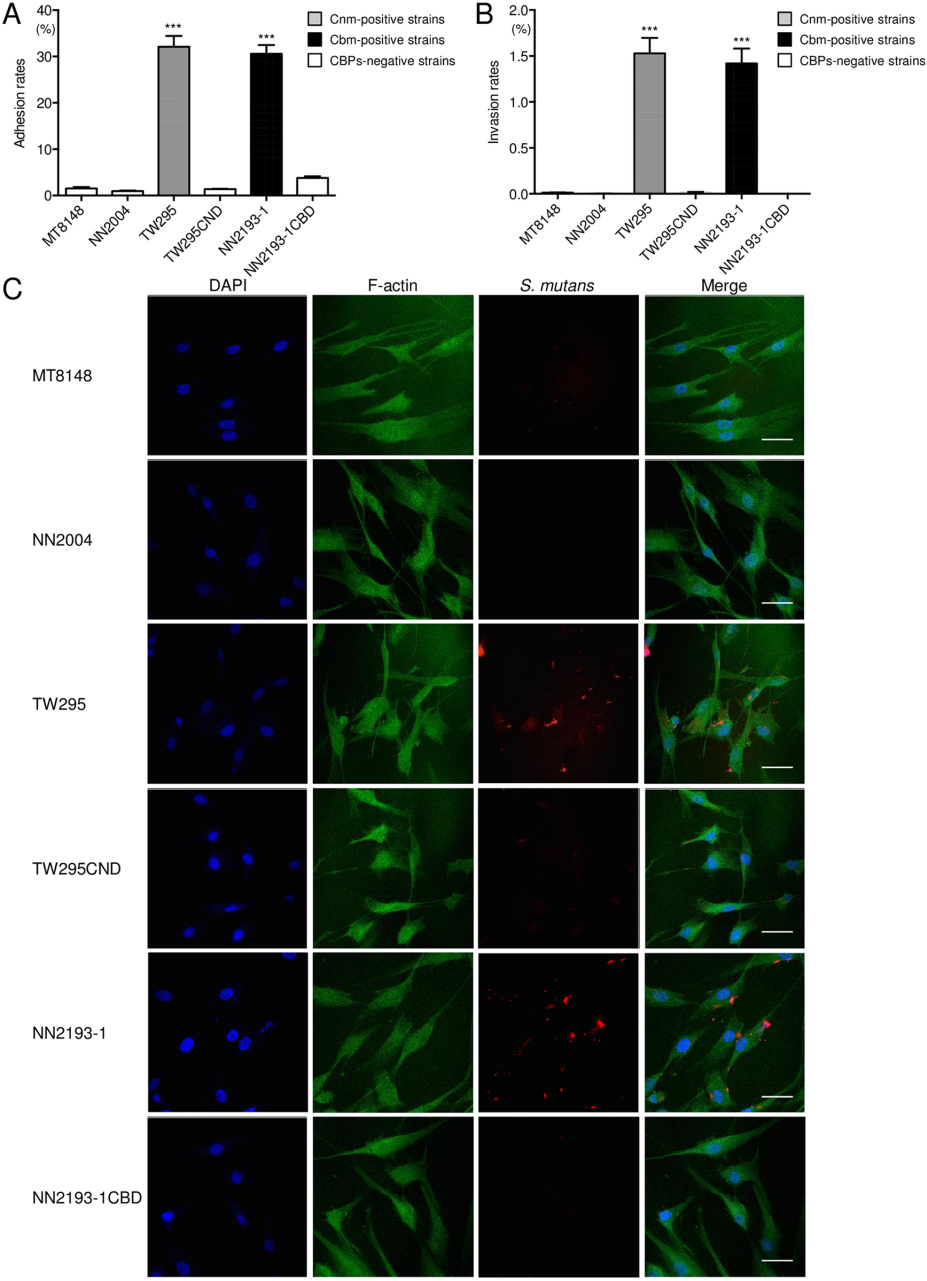
Adhesion and invasion properties of *S*. *mutans* strains with HDPFs. (A) Adhesion to and (B) invasion of HDPFs isolated from a primary tooth. These rates were calculated based on the ratio of recovered infected strains with a multiplicity of infection (MOI) of 100. Data are expressed as the mean ± SD from three independent experiments, with three wells analyzed for each sample. CBP-positive strains showed significant differences compared with CBP-negative strains (***P<0.001). (C) Representative confocal scanning laser microscopic images of HDPFs isolated from primary teeth. Nuclei are stained blue, F-actin is green, and bacterial cells adherent to HDPFs are red. Bars, 40 μm.

The rates of adhesion to and invasion of HDPFs isolated from primary teeth for the TW295 (Cnm-positive) and NN2193-1 (Cbm-positive) strains were significantly higher compared with their Cnm- and Cbm-defective isogenic mutant strains, respectively, and other CBP-negative strains (*P*<0.001; Fig 1A and 1B). Observation by confocal scanning laser microscopy showed that the strains TW295 and NN2193-1 had infected the HDPFs (Fig 1C). However, no Cnm- or Cbm-defective isogenic mutant, or other CBP-negative strains had infected the fibroblasts. Higher adhesion and invasion properties of TW295 and NN2193-1 were also observed following examination of HDPFs isolated from the permanent tooth (S1 Fig).

### Effects of *S*. *mutans* infection on HDPF cells

**Fig 2:**
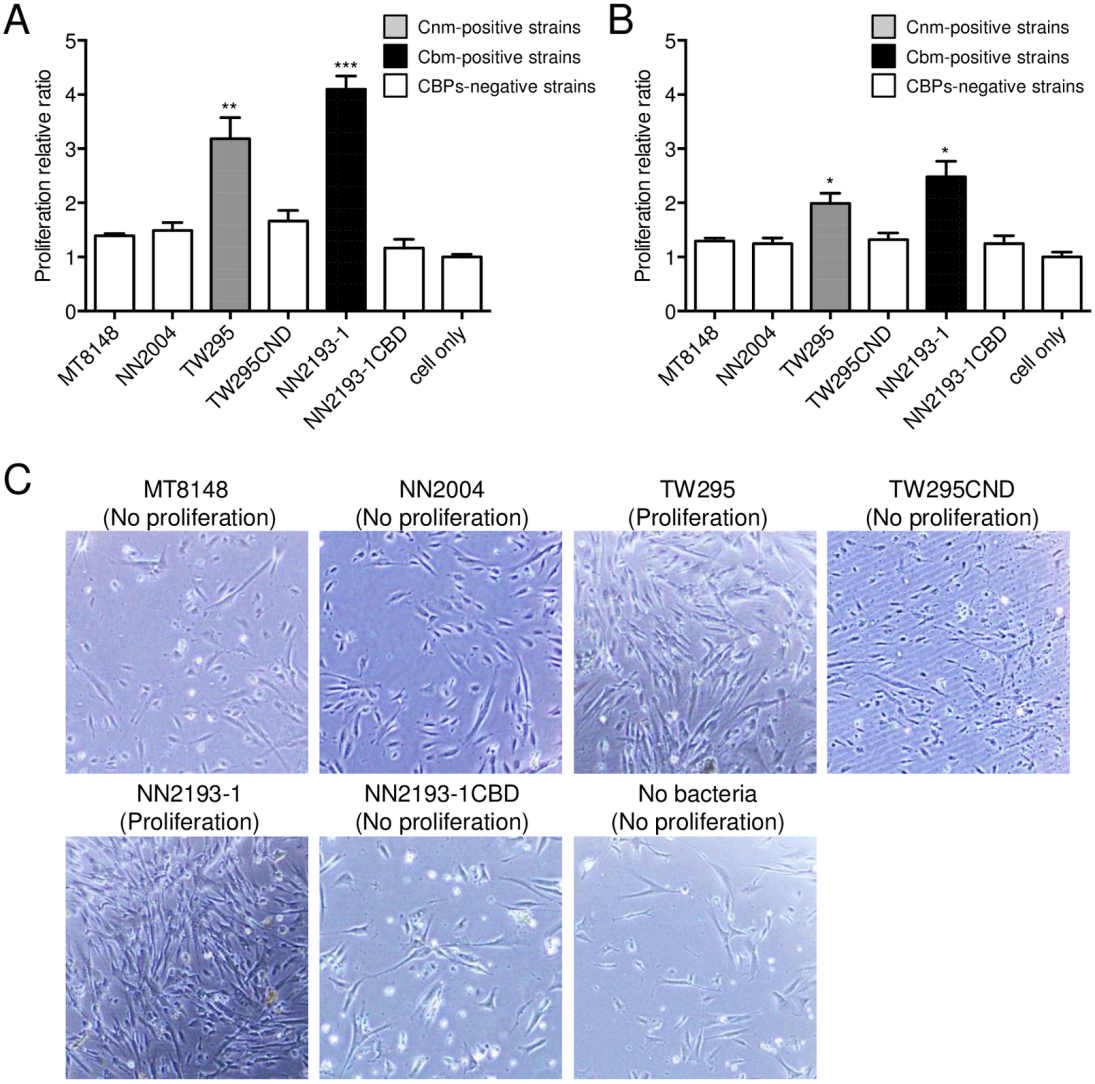
Effects of *S*. *mutans* infection of HDPFs. MTT assay results following infection of HDPFs from a (A) primary and (B) permanent tooth with *S*. *mutans* strains at an MOI of 100 for 6 h. Data are expressed as the relative ratio standardized to non-infected HDPFs and the mean ± SD, with analysis of three independent experiments. CBP-positive strains showed significant differences compared with CBP-negative strains (Student’s *t*-test; *P<0.05, **P<0.01, ***P<0.001). (C) Light microscopy images showing morphology of HDPFs from a primary tooth infected with *S*. *mutans* at an MOI of 100 for 6 h.

Infection with the strains TW295 and NN2193-1 stimulated proliferation of HDPFs isolated from primary teeth. This amount of infection was significantly greater compared with that stimulated by the CBP-defective isogenic mutant and other CBP-negative strains (*P*<0.01 and *P*<0.001, respectively; Fig 2A). CBP-positive *S*. *mutans* strains did not cause prominent proliferation of HDPFs isolated from permanent teeth, although there was significantly greater proliferation in CBP-positive strains compared with CBP-negative strains (*P*<0.05; Fig 2B). When we compared the relative ratios that were obtained in experiments using HDPFs isolated from primary and permanent teeth, those for the TW295 and NN2193-1 strains were significantly higher in cultures with primary teeth specimens than in permanent teeth (*P*<0.05 and *P*<0.01). Light microscopy images showed that infection with TW295 and NN2193-1 induced overgrowth of HDPFs isolated from primary teeth (Fig 2C).

### Frequency of distribution of *S*. *mutans* and serotype classification in bacteria isolated from inflamed dental pulp

**Fig 3:**
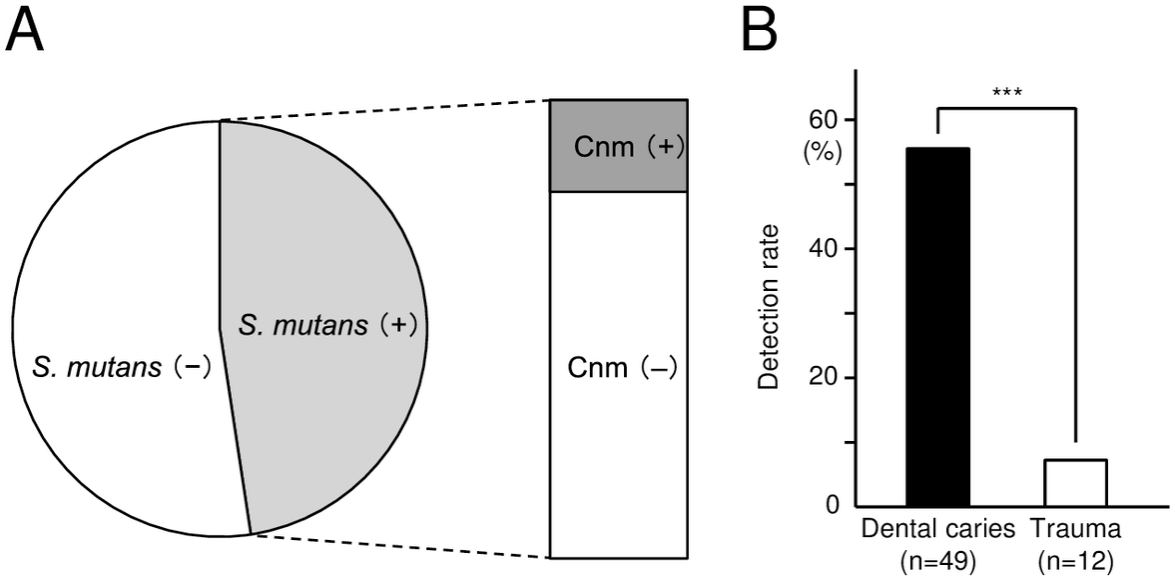
Distribution of *S*. *mutans* in inflamed pulp specimens. (A) Distribution of Cnm-positive and Cnm-negative *S*. *mutans* strains. (B) Detection rates of *cnm*-positive *S*. *mutans* isolated from inflamed pulp induced by dental caries (n = 49) and trauma (n = 12). Significant differences were found (Student’s *t* test; ***P<0.001).

The *S*. *mutans* strains isolated from inflamed dental pulp in the present study are shown in S1 Table. *S*. *mutans* was isolated from 30 (46.9%) of 64 root canal specimens (Fig 3A), all of which were classified as serotype *c* or *e*, with neither serotype *f* nor *k* detected (S1 and S2 Tables). In addition, the detection rate of *S*. *mutans* in inflamed dental pulp caused by development of dental caries was significantly higher than that in inflamed dental pulp caused by trauma (*P*<0.001; Fig 3B).

### Distribution and collagen-binding properties of strains with the *cnm* gene

**Fig 4:**
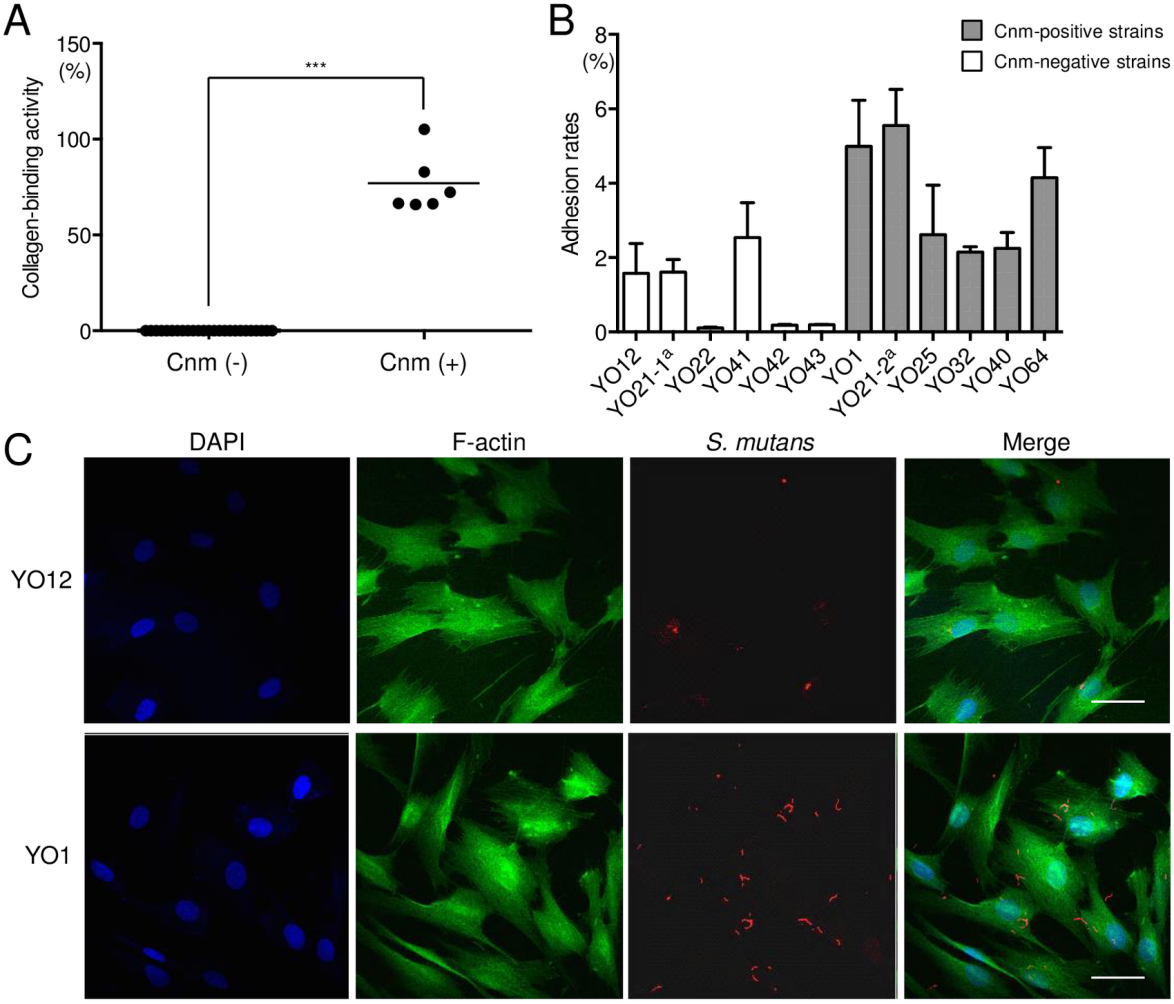
Virulence properties of *S*. *mutans* strains isolated from inflamed pulp specimens. (A) Binding to type I collagen of *S*. *mutans* strains. Significant differences were found (Student’s *t* test; ***P<0.001). (B) Adhesion properties of *S*. *mutans* strains with HDPFs. ^a^YO21-1 and YO21-2 were isolated from the same patient. (C) Representative confocal scanning laser microscopic images of HDPFs isolated from a primary tooth. Nuclei are stained blue, F-actin is green, and bacterial cells adherent to HDPFs are red. Bars, 20 μm.

Of the 30 *S*. *mutans* strains isolated from dental pulp, six were positive for the *cnm* gene (20.0%), whereas none were positive for the *cbm* gene (Fig 3A). One of the six *cnm*-positive specimens (YO21) harbored *cnm*-positive and *cnm*-negative *S*. *mutans* strains (S1 Table). All of the *cnm*-positive *S*. *mutans* strains and none of the *cnm*-negative strains showed collagen-binding properties (Fig 4A).

### Adhesion to and invasion of HDPFs by strains isolated from inflamed pulp

All of the *cnm*-positive *S*. *mutans* strains showed properties of adhesion to HDPFs at a rate >2% (Fig 4B), whereas all of those without the *cnm* gene showed adhesion of <3%. The average rate of adhesion of the *cnm*-positive strains was significantly higher than that of the *cnm*-negative strains (*P*<0.01). Observation by confocal scanning laser microscopy also showed interaction of the *cnm*-positive *S*. *mutans* strain YO1 with HDPFs, whereas few bacterial interactions were observed when the *cnm*-negative *S*. *mutans* strain YO12 was the infecting bacterium (Fig 4C). The average rate of invasion of the *cnm*-positive strains was 0.3%, whereas most of the *cnm*-negative strains had negligible invasion properties, with a significant difference between the groups (*P*<0.01, data not shown).

## Discussion

*S*. *mutans* is a major pathogen of dental caries in humans. Several studies of the cariogenicity of *S*. *mutans* have focused on bacterial cell surface proteins, such as the three types of glucosyltransferases, which synthesize water-insoluble or water -soluble glucans from sucrose [29–31]. Another bacterial cell surface protein is the cell surface 190-kDa protein antigen, known as PA, streptococcal protein antigen P (SpaP), or antigen I/II, and this is correlated with cellular hydrophobicity and sucrose-independent adhesion [32]. Recently, CBPs termed Cnm and Cbm were shown to have a frequency of distribution in oral strains ranging from approximately 10 to 20% [3]. The mechanisms of colonization and pathogenesis in the oral cavity related to CBPs are completely distinct from the other cell surface proteins because CBP-positive strains have an advantage of strong adhesion to collagenous tissue [12]. In the present study, we evaluated the virulence of CBP-positive *S*. *mutans* strains toward pulp cells and demonstrated their presence in inflamed root canals.

Some CBP-positive strains have low cellular hydrophobicity because of a lack of expression of the 190-kDa protein antigen [19], which is considered to be a major virulence factor for the onset of dental caries. In addition, most CBP-positive strains lack the glucan-binding protein GbpA, which is important for adherence to tooth surfaces [18]. CBP-positive *S*. *mutans* strains missing GbpA have lower levels of sucrose-independent and sucrose-dependent adhesion compared with CBP-negative strains [28]. However, Cnm-positive strains show strong adhesion to collagenous hard tissues, such as those of the dentin and root. This suggests that Cnm-positive strains have greater virulence compared with Cnm-negative strains in deep caries, such as dentin caries and areas with pulpitis. A previous study found that clinical parameters indicating dental caries were significantly increased in patients harboring Cnm-positive *S*. *mutans* compared with those with Cnm-negative strains [4].

Among CBP-positive *S*. *mutans* strains, TW295 showed large bacterial mass formation, whereas small bacterial cells of YO1 were widely distributed (S2 Fig), when these strains were infected with HDPFs. In our previous study, TW295 and NN2193-1 did not express the 190-kDa cell surface protein antigen PA [18]. We also previously showed that approximately 25% of the CBP-positive strains lacked PA [18]. However, all CBP-positive strains, including YO1, isolated from inflamed dental pulp in the present study expressed PA (data not shown). The CBP+/PA− strain infected with human vein endothelial cells forms a large bacterial mass, resulting in a high level of adhesion and invasion properties [5, 18]. However, CBP+/PA+ *S*. *mutans* strains do not show such mass formation, resulting in lower adhesion and invasion properties in human vein endothelial cells compared with the CBP+/PA− strain [18]. Although the mechanism of bacterial mass formation remains to be determined, formation of this mass could be related to high virulence in cardiovascular disease [18]. In the present study, we also observed bacterial mass formation in CBP+/PA− *S*. *mutans* strains infecting HDPFs (S2 Fig). To confirm this result, we compared the adhesion properties of *S*. *mutans* TW871 (CBP+), its Cnm-defective mutant TW871CBD [21], and TW871PD, an isogenic mutant of TW871 without PA expression [18], to HDPFs. TW871 showed significantly higher adhesion properties to HDPFs compared with TW871CND (S2 Fig). However, TW871PD showed bacterial mass formation with the highest adhesion properties. Further large-scale studies of *S*. *mutans* clinical strains isolated from inflamed dental pulp should be performed to clarify the frequency of distribution of CBP+/PA− strains in dental pulp specimens.

Proliferation of fibroblast cells is a response to strong inflammation [33]. With regard to proliferation of fibroblast cells in dental pulp, proliferative pulpitis, also named pulp polyps, is an inflammatory type of hyperplasia [34]. In the present study, we found evidence that infection of HDPFs with CBP-positive *S*. *mutans* strains that were isolated from primary and permanent teeth triggered HDPF proliferation. However, such cellular proliferation induced by the CBP-positive *S*. *mutans* was not observed in human vein endothelial cells (data not shown). Among the infected HDPFs, proliferation was more prominently observed in those that were isolated from the primary tooth compared with those that were isolated from the permanent tooth. In our study, the adhesion and invasion rates of CBP-positive strains with HDPFs isolated from the primary teeth were not significantly different compared with those with HDPFs isolated from the permanent teeth. This finding suggests that the distinct proliferation rates of the two cell types may have been owing to differential expression of host molecules, such as fibroblast growth factors. Additional studies are needed to clarify the detailed mechanism of proliferation of HDPFs infected by *S*. *mutans*.

*S*. *mutans* was detected in approximately 30% of cases of dentin caries in direct contact with pulp in a previous study that used molecular analysis [13], similar to the detection rate in our study. In addition, we found that 20% of these bacteria were CBP-positive. All of these bacteria were Cnm-positive, while no Cbm-positive strains were isolated from inflamed pulp, even though the Cbm-positive strain NN2193-1 showed high levels of adhesion to and invasion of HDPFs. A possible explanation for this finding is the relatively low frequency of distribution of Cbm-positive strains in dental plaque or saliva (approximately 2%), whereas Cnm-positive *S*. *mutans* strains showed a distribution of approximately 10–20% [3]. Although the overall distribution rate of Cbm-positive *S*. *mutans* strains is not high, they are important for development of cardiovascular diseases because of their ability to induce platelet aggregation in the presence of fibrinogen [35]. Large-scale studies of *S*. *mutans* clinical isolates obtained from several hundred subjects should be performed to clarify the distribution of Cbm-positive *S*. *mutans* in inflamed pulp.

*S*. *mutans* is involved in infective endocarditis. We have investigated the relationship of CBP-positive *S*. *mutans* strains with the pathogenicity of cardiovascular diseases. We have reported that CBP-positive *S*. *mutans* strains may also be an important virulence factor in other systemic diseases, such as hemorrhagic stroke, ulcerative colitis, non-alcoholic fatty liver disease, and IgA nephropathy [6–9]. The initial step in these systemic diseases is invasion of the bloodstream by *S*. *mutans* [16]. Bacteremia can be caused by invasive dental treatments, such as tooth extraction, scaling, and root planning, as well as conservative dental procedures [16, 36]. A recent report showed that *S*. *mutans* was detected in carious dentin in direct contact with irreversible pulpitis at a high frequency [13]. In the present study, *S*. *mutans* organisms were isolated from infected root canals in direct contact with the vascular system, indicating that the canal is a bacterial reservoir, resulting in frequent occurrence of bacteremia. Therefore, deleterious habits, such as increased sucrose consumption or poor oral hygiene leading to severe dental caries, may facilitate infection of CBP-positive *S*. *mutans* strains into the bloodstream.

In summary, CBP-positive *S*. *mutans* strains show adhesion to and invasion of HDPFs, and this induces proliferation of these cells in primary tooth cultures. Furthermore, *S*. *mutans* strains are present in inflamed pulp at a high frequency, of which approximately 20% are Cnm-positive. Our findings indicate that the presence of CBP-positive *S*. *mutans* in root canals as an endodontic pathogen may be a risk factor for bacteremia, especially during root canal treatment.

## Supporting Information

S1 Fig Rates of (A) adhesion and (B) invasion of *S*. *mutans* in HDPFs isolated from a permanent tooth. Rates were calculated based on the ratio of recovered/infected strains with a multiplicity of infection of 100. Data are expressed as the mean ± SD from three independent experiments, with three wells analyzed for each sample. CBP-positive and CBP-negative strains showed significant differences (***P<0.001).
(PDF)

S2 Fig Adhesion properties of CBP+/PA+ and CBP+/PA− *S*. *mutans* strains with HDPFs. Representative confocal scanning laser microscopic images of HDPFs isolated from primary teeth infected with (A) YO1 and TW295, and (B) TW871 and their isogenic mutant strains. Nuclei are stained blue, F-actin is green, and bacterial cells adherent to HDPFs are red. Bars, 20 μm. (C) Adhesion properties of TW871 and isogenic mutant strains to HDPFs isolated from a primary tooth. The adhesion rates were calculated based on the ratio of recovered infected strains with a multiplicity of infection (MOI) of 100. Data are expressed as the mean ± SD from three independent experiments, with four wells analyzed for each sample. There were significant differences between the parent strains and isogenic mutants (**P<0.01; ***P<0.001).
(PDF)

S1 Table Summary of *S*. *mutans* isolated from severe dental caries in 49 subjects. (DOC)

S2 Table Summary of *S*. *mutans* isolated from teeth with trauma or fracture of the central cusp in 15 subjects. (DOCX)
