# Supplementary figures and images for: Contribution of the Collagen-Binding Proteins of Streptococcus mutans to Bacterial Colonization of Inflamed Dental Pulp
Source: PLoS One. 2016 Jul 21;11(7):e0159613. doi: 10.1371/journal.pone.0159613 (PMC4956251; doi:10.1371/journal.pone.0159613)

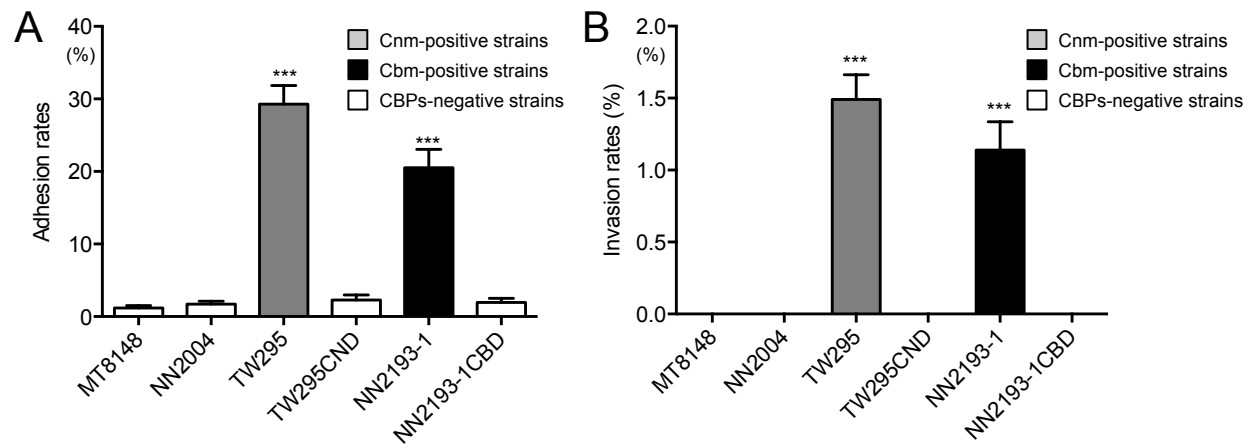

**S1 Fig**

Supplement: S1 Fig — Rates were calculated based on the ratio of recovered/infected strains with a multiplicity of infection of 100. Data are expressed as the mean ± SD from three independent experiments, with three wells analyzed for each sample. CBP-positive and CBP-negative strains showed significant differences (***P<0.001). (PDF) [file pone.0159613.s001.pdf]

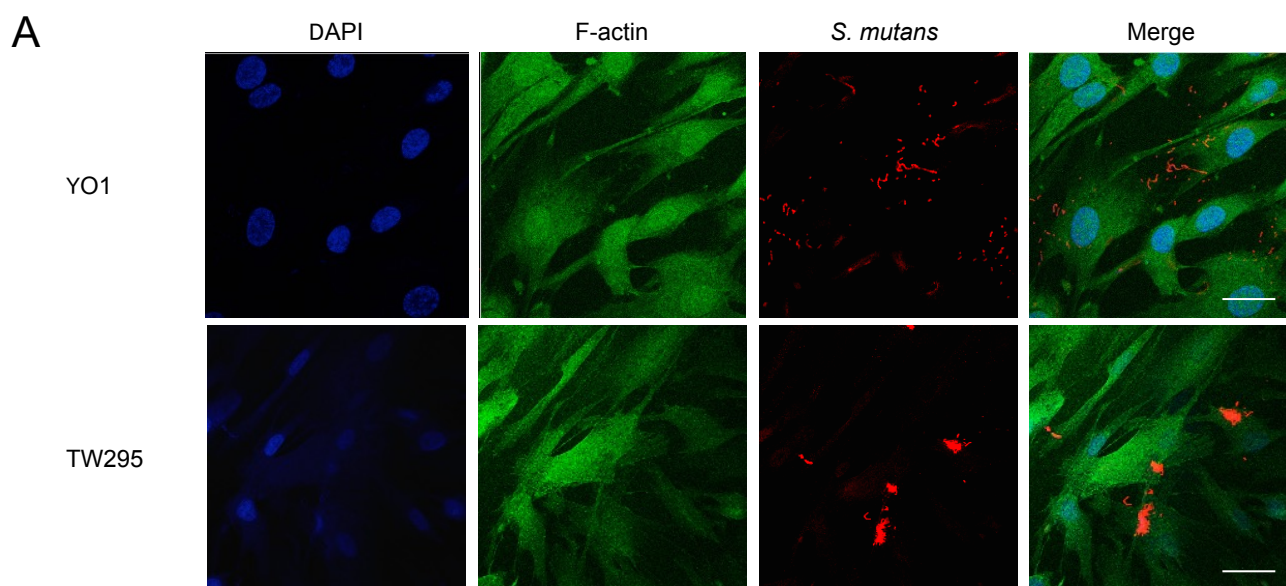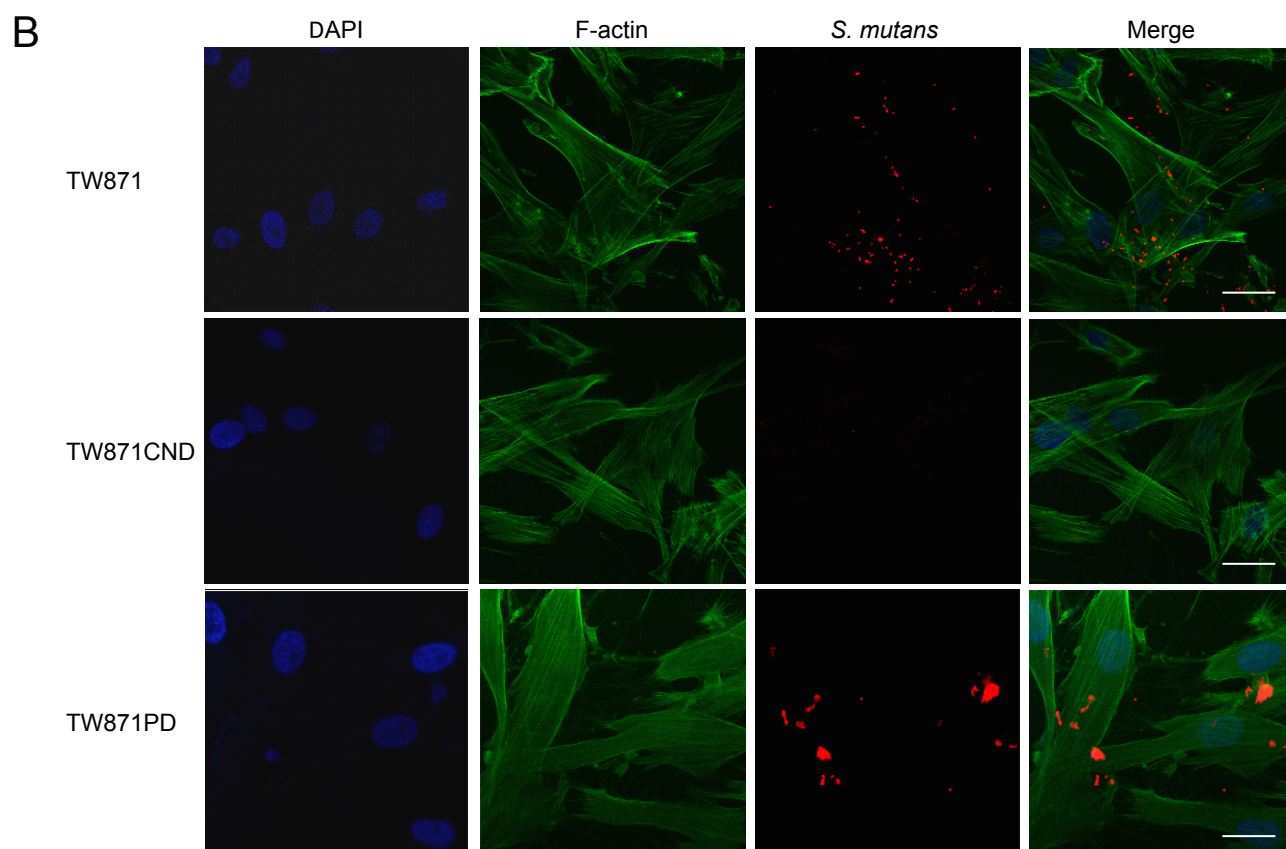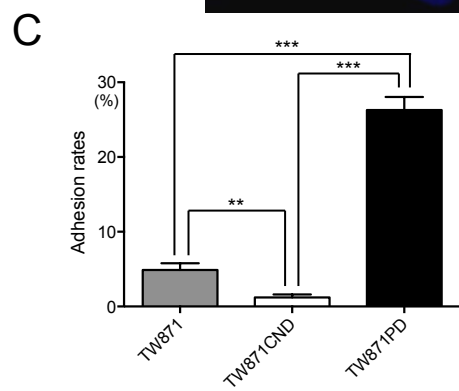

**S2 Fig**

Supplement: S2 Fig — Representative confocal scanning laser microscopic images of HDPFs isolated from primary teeth infected with (A) YO1 and TW295, and (B) TW871 and their isogenic mutant strains. Nuclei are stained blue, F-actin is green, and bacterial cells adherent to HDPFs are red. Bars, 20 μm. (C) Adhesion properties of TW871 and isogenic mutant strains to HDPFs isolated from a primary tooth. The adhesion rates were calculated based on the ratio of recovered infected strains with a multiplicity of infection (MOI) of 100. Data are expressed as the mean ± SD from three independent experiments, with four wells analyzed for each sample. There were significant differences between the parent strains and isogenic mutants (**P<0.01; ***P<0.001). (PDF) [file pone.0159613.s002.pdf]
