# Supplementary material for: Contribution of the Collagen-Binding Proteins of Streptococcus mutans to Bacterial Colonization of Inflamed Dental Pulp
Source: PLoS One. 2016 Jul 21;11(7):e0159613. doi: 10.1371/journal.pone.0159613 (PMC4956251; doi:10.1371/journal.pone.0159613)
Supplement: S1 Table — (DOC) [file pone.0159613.s003.doc]

S1 Table

Summary of *S. mutans* isolated from severe dental caries in 49 subjects.

|  |  |  |  |  | | |  | | |
| --- | --- | --- | --- | --- | --- | --- | --- | --- | --- |
|  |  |  |  | | | PCR | | | |
| Subjects | Age | Gender | Location | | *S. mutans* | | | Cnm | Serotype |
|  |  |  |  | |  | | |  |  |
|  |  |  |  | |  | | |  |  |
| YO1 | 3Y11M | Female | ULD | | + | | | + | *e* |
| YO2 | 3Y11M | Male | LLD | | + | | | − | *e* |
| YO3 | 3Y9M | Male | LRE | | + | | | − | *c* |
| YO4 | 4Y0M | Male | LLE | | + | | | − | *c* |
| YO5 | 4Y4M | Male | LLD | | − | | |  |  |
| YO6 | 4Y7M | Female | LLCDE | | − | | |  |  |
| YO7 | 4Y7M | Male | ULA | | − | | |  |  |
| YO8 | 4Y9M | Female | URA | | + | | | − | *c* |
| YO9 | 4Y9M | Female | ULA | | + | | | − | *c* |
| YO10 | 5Y3M | Female | LLD | | + | | | − | *c* |
| YO11 | 5Y4M | Male | ULE | | − | | |  |  |
| YO12 | 5Y5M | Male | ULD | | + | | | − | *e* |
| YO13 | 5Y5M | Male | URE | | + | | | − | *e* |
| YO14 | 5Y5M | Male | URE | | − | | |  |  |
| YO15 | 5Y7M | Male | ULD | | + | | | − | *c* |
| YO16 | 5Y9M | Female | LLD | | − | | |  |  |
| YO17 | 6Y0M | Male | LLD | | − | | |  |  |
| YO18 | 6Y10M | Female | ULE | | − | | |  |  |
| YO19 | 6Y11M | Male | LLD | | + | | | − | *c* |
| YO20 | 6Y11M | Female | LRD | | − | | |  |  |
| YO21 | 6Y11M | Male | LRE | | + | | | +^*^ | *c* |
| YO22 | 6Y2M | Female | URD | | + | | | − | *e* |
| YO23 | 6Y9M | Male | LRD | | + | | | − | *c* |
| YO24 | 7Y1M | Male | LLE | | − | | |  |  |
| YO25 | 7Y11M | Male | LRE | | + | | | + | *c* |
| YO26 | 7Y11M | Male | LRD | | − | | |  |  |
| YO27 | 7Y2M | Female | LRD | | − | | |  |  |
| YO28 | 7Y4M | Female | ULD | | + | | | − | *c* |
| YO29 | 7Y4M | Female | LRD | | + | | | − | *c* |
| YO30 | 7Y4M | Male | LLE | | − | | |  |  |
| YO31 | 7Y5M | Female | URD | | − | | |  |  |
| YO32 | 8Y0M | Male | ULE | | + | | | + | *c* |
| YO33 | 8Y6M | Male | LRD | | − | | |  |  |
| YO34 | 8Y1M | Male | LLE | | − | | |  |  |
| YO35 | 8Y2M | Male | URD | | + | | | − | *c* |
| YO36 | 8Y6M | Male | LLD | | − | | |  |  |
| YO37 | 9Y11M | Male | LL6 | | − | | |  |  |
| YO38 | 9Y2M | Male | ULE | | − | | |  |  |
| YO39 | 9Y2M | Male | ULD | | − | | |  |  |
| YO40 | 9Y5M | Female | LRE | | + | | | + | *e* |
| YO41 | 10Y2M | Male | LRD | | + | | | − | *e*/*c* |
| YO42 | 10Y6M | Female | LRE | | + | | | − | *c* |
| YO43 | 10Y8M | Female | LL3 | | + | | | − | *c* |
| YO44 | 11Y1M | Male | LLE | | − | | |  |  |
| YO45 | 11Y4M | Female | LL1 | | + | | | − | *c* |
| YO46 | 13Y6M | Female | UR1 | | − | | |  |  |
| YO47 | 14Y10M | Male | LL1 | | + | | | − | *c* |
| YO48 | 15Y10M | Male | ULE | | + | | | − | *c* |
| YO49 | 20Y0M | Female | UR8 | | + | | | − | *c* |

* 3 of 5 *S. mutans* strains showed Cnm positive

URD; upper right primary first molar, URE; upper right primary second molar, ULD; upper left primary first molar, LLD; lower left primary first molar, ULE; upper left primary second molar, LRE; lower right primary second molar, LLE; lower left primary second molar, LRD; lower right primary first molar, LRE; lower right primary second molar, URA; upper right primary central incisor, ULA; upper left primary central incisor, LLC; lower left primary canine, UR1; upper right central incisor, LL1; lower left central incisor, LL3; lower left canine, LL6; lower left first molar, UR8; upper right third molar
