# Supplementary material for: Contribution of the Collagen-Binding Proteins of Streptococcus mutans to Bacterial Colonization of Inflamed Dental Pulp
Source: PLoS One. 2016 Jul 21;11(7):e0159613. doi: 10.1371/journal.pone.0159613 (PMC4956251; doi:10.1371/journal.pone.0159613)
Supplement: S2 Table — (DOCX) [file pone.0159613.s004.docx]

S2 Table

Summary of *S. mutans* isolated from teeth with trauma or fracture of central cusp in 15 subjects.

|  |  |  |  |  | | |  | | |
| --- | --- | --- | --- | --- | --- | --- | --- | --- | --- |
|  |  |  |  | | | PCR | | | |
| Subjects | Age | Gender | Location | | *S. mutans* | | | Cnm | Serotype |
|  |  |  |  | |  | | |  |  |
|  |  |  |  | |  | | |  |  |
| YO50 | 1Y8M | Female | ULA | | − | | |  |  |
| YO51 | 3Y8M | Male | URA | | − | | |  |  |
| YO52 | 4Y6M | Male | ULA | | − | | |  |  |
| YO53 | 5Y2M | Male | ULA | | − | | |  |  |
| YO54 | 7Y10M | Male | LLC | | − | | |  |  |
| YO55 | 8Y2M | Female | UL1 | | − | | |  |  |
| YO56 | 8Y9M | Female | UR1 | | − | | |  |  |
| YO57 | 9Y0M | Female | UL1 | | − | | |  |  |
| YO58 | 10Y5M | Male | LR5 | | + | | | − | *c* |
| YO59 | 10Y7M | Female | UR1 | | − | | |  |  |
| YO60 | 11Y5M | Female | LL5 | | − | | |  |  |
| YO61 | 12Y6M | Male | UR1 | | − | | |  |  |
| YO62 | 13Y6M | Female | LR5 | | + | | | − | *c* |
| YO63 | 19Y0M | Male | UR1 | | − | | |  |  |
| YO64 | 19Y0M | Male | UR1 | | + | | | + | *c* |

URA; upper right primary central incisor, ULA; upper left primary central incisor, LRD; lower right primary first molar, LRE; lower right primary second molar, LLC; lower left primary canine, UR1; upper right central incisor, UL1; upper left central incisor, LR5; lower right first premolar, LL5; lower left first premolar.
